# Analysis of the difference between early-bolting and non-bolting roots of *Angelica dahurica* based on transcriptome sequencing

**DOI:** 10.1038/s41598-023-34554-5

**Published:** 2023-05-15

**Authors:** Ping Wu, Xiaoyu Wang, Junxia Guo, Songli Zhang, Qingmiao Li, Mei Zhang, Qingmao Fang, Bin Luo, Hongsu Wang, Weijin He

**Affiliations:** Sichuan Academy of Traditional Chinese Medicine Sciences, Sichuan Genuine Medicinal Materials System Development Engineering Technology Research Center, Sichuan Key Laboratory of Quality and Innovation of Traditional Chinese Medicine, Chengdu, 610041 China

**Keywords:** Transcriptomics, Plant molecular biology, Plant morphogenesis

## Abstract

*Angelica dahurica* (Fisch. ex Hoffm.) Benth.et Hook.f.var.*formosana* (Boiss.) Shan et Yuan (*A. dahurica*) is a well-known medicinal plant that has a wide range of applications in the pharmaceutical, food, cosmetic, and other industries. However, the issue of early bolting has emerged as a major hindrance to its production. This problem not only reduces the yield of *A. dahurica*, but also has an impact on its active ingredients. To date, the molecular factors that contribute to early bolting and its impact on the growth of *A. dahurica* have not been thoroughly investigated. Therefore, we conducted a transcriptome study using the Illumina NovaSeq 6000 on two developmental types: early-bolting and non-bolting (normal) roots of *A. dahurica*. We obtained 2,185 up-regulated and 1,414 down-regulated genes in total. Many of the identified transcripts were related to genes involved in early bolting. The gene ontology analysis revealed several differentially expressed genes that are crucial in various pathways, primarily associated with cellular, molecular, and biological processes. Additionally, the morphological characteristics and coumarin content in the early bolting roots of *A. dahurica* were significantly altered. This study provides insight into the transcriptomic regulation of early bolting in *A. dahurica,* which can potentially be utilized to enhance its medicinal properties.

## Introduction

*A. dahuricae* is a traditional Chinese medicinal herb that belongs to the Umblliferae family^1^. Its dried roots have been commonly used in traditional Chinese medicine to alleviate exterior cold, dispel wind and pain, eliminate swelling, and discharge pus^2^. *A. dahurica* is a well-known Chinese herbal medicine that is used both medicinally and as a food source in China. This multi-purpose plant has a wide range of applications, including as a raw material for producing medicines, health products, and skin care items, making it a valuable commodity in the market^3^. At present, the majority of *A. dahuricae* is cultivated rather than harvested from the wild. Due to its adaptability, this plant is grown in many regions across China, and has a long history of cultivation. However, the continued development of the *A. dahuricae* industry is hindered by early bolting, a phenomenon where the plant prematurely transitions from vegetative growth to reproductive growth phase, ultimately leading to early fowering and seed set.

Early bolting greatly affects the yield and quality of medicinal plants^4^. When *A. dahurica* begins to bolt and flower, nutrients are redirected from the root to the floral shoot. This results in a decrease in the accumulation of secondary metabolites and a reduction in the medicinal and nutritional value of the root^5^. Therefore, it is crucial to prevent early bolting in order to improve the quality and yield of *A. dahurica*. Previous investigations aimed at understanding and reducing early bolting in *A. dahurica* have primarily focused on physiological and cultivation aspects^6,7^. However, research on the molecular aspects of early bolting in *A. dahurica* is just beginning. Early bolting in *A. dahuricae* involves several gene families, including MYB-related, NAC, and CONSTANS-like genes. That are associated with secondary cell wall formation, transcription factors(TFs), and flowering regulation^8–10^. Despite some reports providing evidence on the molecular mechanisms underlying early bolting in *A. dahuricae*, the related genes remain largely unknown.

RNA-sequencing (RNA-seq) technology is a valuable tool that can be applied to study the transcriptome of non-reference genome organisms^11^. It plays a crucial role in mining functional genes^12–14^, analyzing developmental mechanisms^15–17^, developing molecular markers^18^, and constructing gene regulatory networks^19–21^.

**Figure 1:**
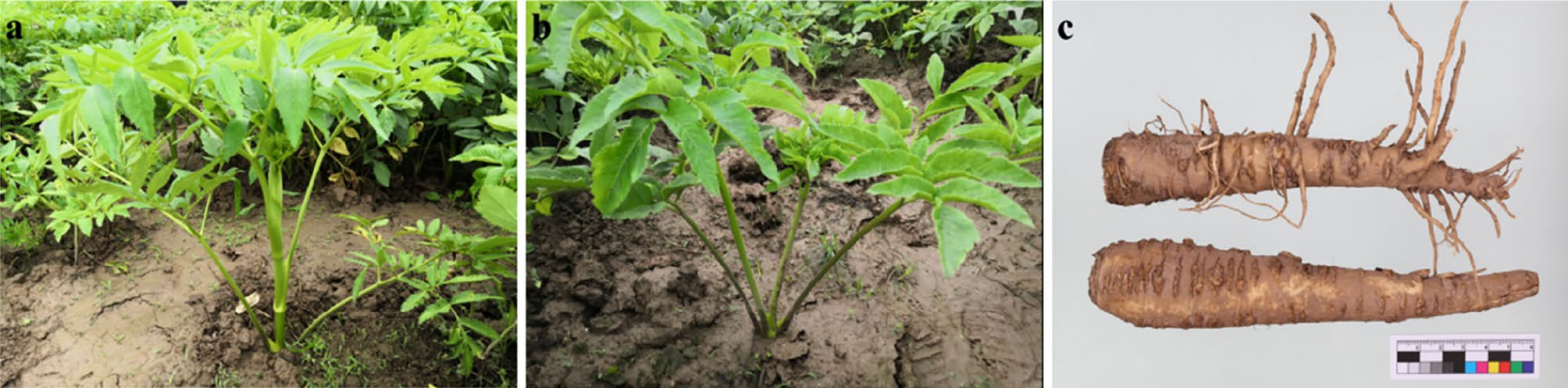
*A. dahurica* test material. (**a**) Images of early-bolting *A. dahurica*(EB) whole plant. (**b**) Images of non-bolting *A. dahurica *(NB) whole plant. (**c**) Images of *A. dahurica* roots. Above is the root of the EB plants, below is the root of the NB plants.

The objective of this study is to obtain a comprehensive global expression profile of the molecular factors that play a crucial role in early bolting, coumarin and lignin metabolism, and other related processes in *A. dahuricae*. In order to investigate the differences in the transcriptome of *A. dahuricae* between an early bolting genotype and a normal genotype (Fig. 1), we have utilized RNA-sequencing technology on the Illumina platform to generate and analyze transcriptomic data.

## Results

### Morphological characteristics

The root diameter, fresh weight, dry weight, and drying rate of NB were found to be significantly greater than those of EB (*p* < 0.05). However, there was no significant difference in root length between EB and NB (Table 1).

**Table 1:** The morphological characteristics from EB and NB.

Item	EB	NB
Root length (cm)	22.0 ± 1.1^a^	21.8 ± 1.1^a^
Root diameter (mm)	27.1 ± 1.4^b^	33.2 ± 1.9^a^
Root fresh weight (g)	62.9 ± 3.1^b^	150.4 ± 7.4^a^
Root dry weight (g)	17.4 ± 0.9^b^	47.4 ± 2.4^a^
Root drying rate (%)	27.7 ± 0.1^b^	31.5 ± 0.1^a^

Data were mean ± SE from biological experiments (n = 9). Data with the different letters were significantly different by Duncan’s multiple range test at *P* < 0.05.

### Coumarins content in EB and NB

**Figure 2:**
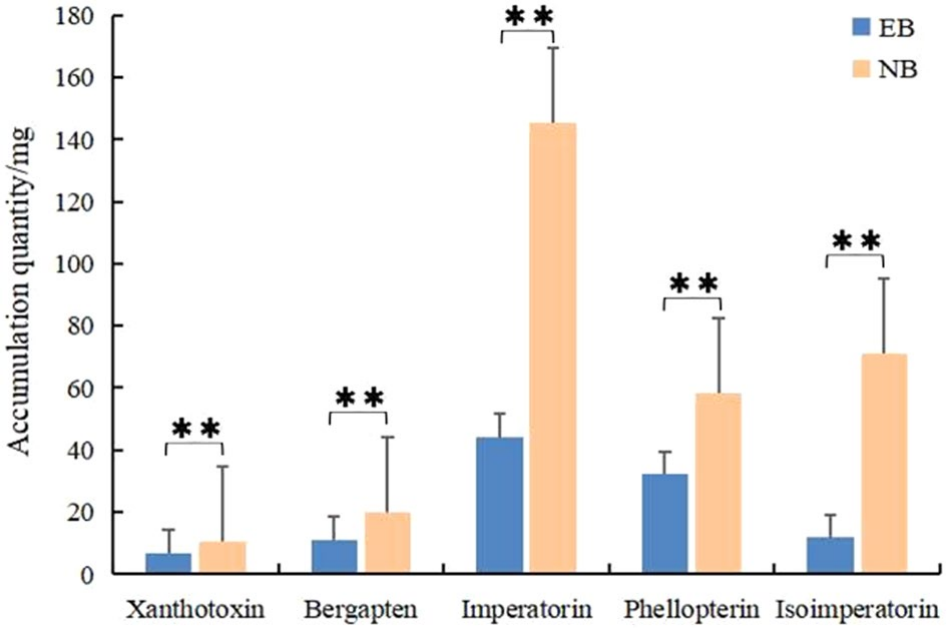
The coumarins accumulation quantity in root of EB and NB. **Means differed extremely significantly (*P* < 0.01).

This study aimed to determine the coumarins content in the roots of *A. dahurica *(Fig. 2). Specifically, we measured the levels of xanthotoxin, bergapten, imperatorin, phellopterin, and isoimperatorin. The results showed that imperatorin had the highest content, accounting for 41.62% in EB and 47.55% in NB. Furthermore, we found that the accumulation quantity of five coumarins were significantly higher in NB compared to EB (*p* < 0.01).

### RNA-Seq and de novo assembly of the *A. dahurica* transcriptome

In this study, we used high throughput RNA-Seq to analyze transcript libraries from the roots of *A. dahurica*. Each sample yielded an average of 22,638,100 reads in EB and 20,989,032 in NB samples from two cDNA libraries. The mapped reads were 18,093,792 and 17,451,497, respectively. The GC content was found to be greater than 43.16%, and the Q30 percentage exceeded 94.40% (Table 2). After using Trinity 2.4.0 software for de novo assembly^22^, we obtained a total of 53,304 unigenes. The N50 length was 1532 bp, and 56.57% of the unigenes were over 500 bp in length, while 29.04% exceeded 1,000 bp (Supplementary Fig. S1). These results indicate that the sampling of *A. dahurica* in our study was reliable and suitable for further analysis.

**Table 2:** RNA sequencing statistics of EB and NB.

Sample name	Total reads	Mapped reads	Uniq mapped reads	Multi mapped reads	Base number	GC content	% ≥ Q30
EB	22,638,100	18,093,792	5,055,281	13,038,511	6,733,316,553	43.16	94.40
NB	20,989,032	17,451,497	5,183,956	12,267,541	6,270,563,213	43.47	94.94

### Functional annotation and classification of ***A. dahurica*** unigenes

In this paper, we compared the unigene sequences of *A. dahurica* samples with a common functional database. In total, 29,401 unigenes were annotated by nine databases, including COG, GO, KEGG, KOG, Pfam, Swiss-Prot, TrEMBL, eggNOG, and Nr (Supplementary Table S1). Of these, 28,803 unigenes were annotated to the Nr database. Interestingly, we found that the *A. dahurica* transcripts showed a high similarity to those of *Daucus carota* (22,985, 79.80%) (Supplementary Fig. S2).

In this study, we annotated 6,259 unigenes of *A. dahurica* across 26 COG pathways (Supplementary Fig. S3). Of these, 688(11.13%) unigenes were classified under translation, ribosomal structure, and biogenesis, while 650(10.52%) unigenes were classified under posttranslational modification, protein turnover, and chaperones. Additionally, we annotated 22,530 unigenes in the GO database (Supplementary Fig. S4), with the most represented functions being binding, cellular anatomical entity, cellular process, metabolic process, and catalytic activity.

Unigenes from *A. dahurica* were compared to the standard metabolic pathways in the KEGG database. The analysis resulted in the successful annotation of 17,895 unigenes into 137 metabolic pathways. The plant-pathogen interaction pathway (ko04626) had the highest number of enriched unigenes (689, 4.76%), followed by the plant hormone signal transduction pathway (ko04075) with 496 unigenes (3.43%) (Supplementary Table S2).

### Identification of Diferential Expression genes(DEGs)

**Figure 3:**
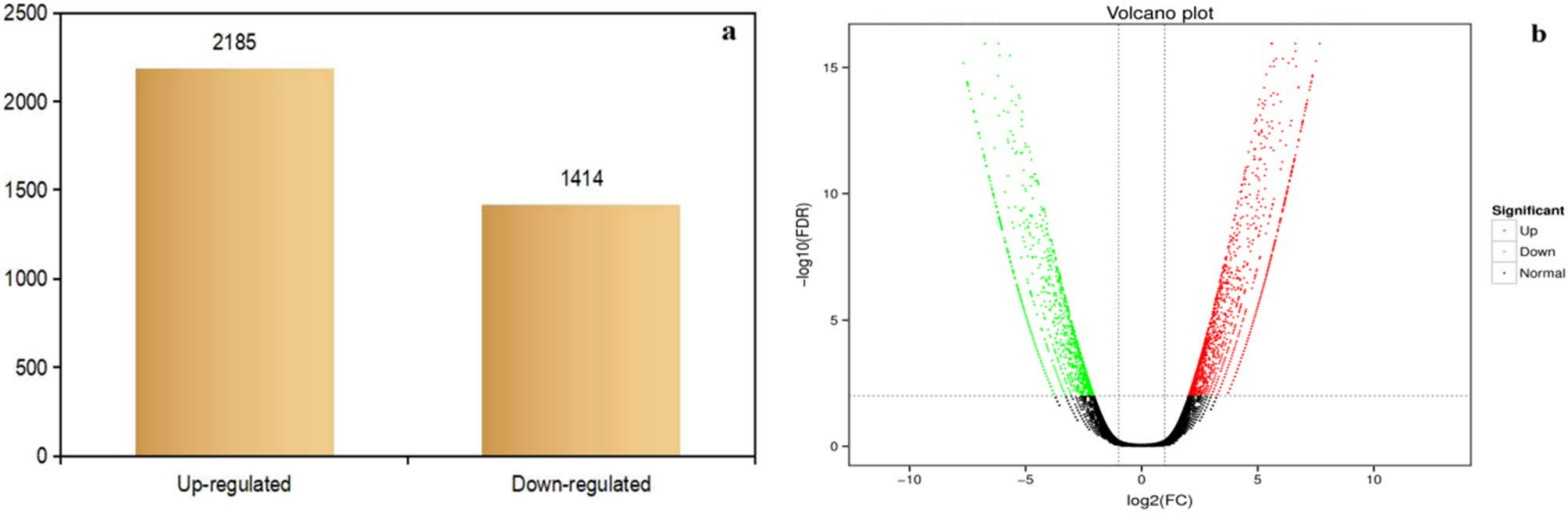
General features of *A. dahurica* transcriptome. (**a**) Total number of DEGs with FDR (false discovery rate) < 0.01 and a FC (fold change) ≥ 2. (**b**) Volcano plot of all differentially expressed genes.

To evaluate diferential gene expression levels in response to early bolting, two groups of EB and NB Illumina clean reads were taken to assemble with the transcriptome. Fragments per kilobase per million reads (FPKM) values of assembling unigenes were calculated with FDR (false discovery rate) < 0.01 and a FC (fold change) ≥ 2. In total, 3,599 DEGs were identified between the EB and NB groups. Of these, 2,185 genes were up-regulated, while 1,414 genes were down-regulated (Fig. 3a). The volcano plot in Fig. 3b displays the expression levels of the genes in the EB and NB groups. The plot uses different colors to indicate up-regulated genes, down-regulated genes, and genes whose expression was not affected.

To determine the biological functions of the DEGs, functional enrichment analysis was conducted. A total of 2,123 DEGs were classifed into 47 functional groups using GO assignments (Supplementary Fig. S5). Of these, 18 functional groups were involved in biological processes, 16 in cellular components, and 13 in molecular functions. In the biological process groups, the metabolic process had the largest enrichment with 978 DEGs (46.07%), followed by cellular process with 839 DEGs (39.52%), and single-organism process with 558 DEGs (26.28%). In the analysis of molecular function processes, the two largest functional groups consisted of 1,096 DEGs (51.63%) assigned to binding and 967 DEGs (45.55%) assigned to catalytic activity. Regarding cellular component domain, approximately 30.33% of DEGs (644 total) were assigned to the membrane, while 28.02% (595 DEGs), 25.95% (551 DEGs), and 25.95% (551 DEGs) were assigned to membrane part, cell, and cell part, respectively.

Furthermore, 864 DEGs were successfully annotated to 127 KEGG pathways to further characterize the molecular functions and biological pathways. A KEGG scatter plot was shown in Supplementary Fig. S6. Overall, these findings shed light on the regulatory elements involved in the early bolting process of *A. dahurica* and will aid in deciphering the functions of these genes.

## Analysis of DEGs

### DEGs associated with early bolting

Several DEGs involved in biochemical and physiological pathways have been identified as being associated with early bolting (Supplementary Table S3). Specifically, genes involved in plant hormone signaling pathways, such as Auxin-induced (*AUX6B*), Auxin-responsive (*IAA26*, *SAUR32*, *SAUR36*, *SAUR61*, *SAUR67*, *SAUR72*), and Gretchen Hagen 3 (*GH3.1*) were found to be up-regulated in EB^23–27^. In contrast, Ethylene-responsive transcription factor (*ERF13, RAP2-7*), AP2/ERF and B3 domain-containing transcription factor (*RAV1*), Auxin-responsive (*IAA14*, *IAA27*, *SAU40*, *SAU76*), Myelocytomatosis genes (*MYC2*, *MYC3)*, and Gibberellin-regulated (*GASA1*, *GASA11*, *GASA14*) genes were all down-regulated^28–34^.

In this study, it was discovered that genes involved in hormone synthesis pathways, including 9-*cis*-epoxycarotenoid dioxygenase (*NCED2*), Abscisic acid 8'-hydroxylase (*ABAH2*), and Cytokinin dehydrogenase genes (*CKX7*, *CKX1*, *CKX6*) were up-regulated. Additionally, Agamous-like MADS-box genes (*AGL8*, *AGL62*, *AP1*), which are involved in floral organ development, were also up-regulated.

The main flowering controlling pathways were found to involve key genes in photoperiodic, vernalization, and gibberellin pathways. Specifically, MADS-box (*SOC1*), Zinc finger protein CONSTANS-LIKE (*COL7*), Flowering locus T (*HD3A*), B3 domain-containing transcription factor (*VRN1*), Gibberellin 20 oxidase (*GA20OX1*, *GA20OX2*), and Gibberellin 2-beta-dioxygenase (*GA2OX5*) were up-regulated in EB. On the other hand, Zinc finger protein CONSTANS-LIKE (*COL5*, *COL13*), FT-interacting (*FTIP1*), B-box domain (*MIP1A*, *MIP1B*), Myb family transcription factor (*EFM*), Cyclic dof factor (*CDF2*), DELLA (*GAIP*), and Gibberellin 2-beta-dioxygenase (*GA2OX1*, *GA2OX6*) were all down-regulated.

### DEGs associated with the biosynthesis of coumarin metabolism

The genes responsible for the biosynthesis of coumarin metabolism (Supplementary Table S2), such as 4-coumarate–CoA ligase *(4CL*), Caffeic acid 3-*O*-Methyltransferase (*COMT*), Shikimate O-hydroxycinnamoyltransferase(*HCT*), Caffeoylshikimate esterase(*CSE*) were found to be down-regulated^35^.

### DEGs associated with the biosynthesis of lignin metabolism

Genes that play a role in lignin metabolism (Supplementary Table S2), including ABC transporter G family members (*ABCG22*, *ABCG36*) were up-regulated, while transcription factors(*MYB1*, *MYB63*) were down-regulated.

### Gene expression changes analysis by qRT-PCR

**Figure 4:**
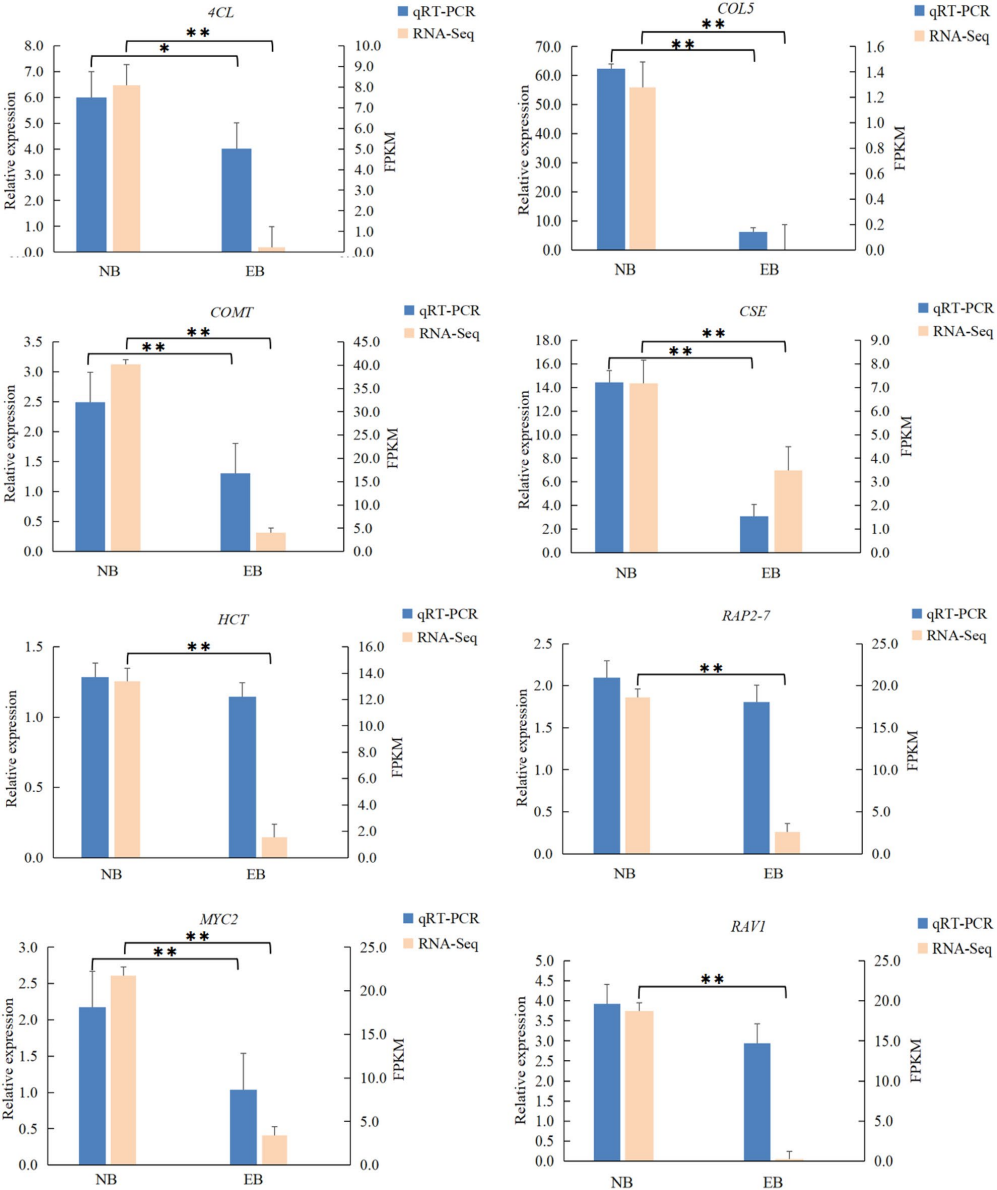
Validation of the expresstion profile of RNA-Seq(FPKM) by qRT-PCR. Eight genes were selected and validated by qRT-PCR to confirm their expresstion profiles determined by RNA-Seq. * Means differed significantly (*P* < 0.05), **Means differed extremely significantly (*P* < 0.01).

To confirm the results of DGEs and RNA-Seq results, qRT-PCR was applied to analyze the expression of eight genes in the roots of *A. dahurica*. All the genes exhibited a comparable trend of expression in EB and NB as attained by transcriptomic data. The expression levels of all eight genes in EB and NB are shown in Fig. 4.

## Discussion

*A. dahurica* is a famous traditional herbal medicine in China. However, its early bolting has severely limited its usage. Early bolting not only reduces the production of *A. dahurica* but also affects the active ingredients in its roots, resulting in a complete loss of its medicinal value. Moreover, the molecular genetic background of *A. dahurica* is still unclear, which further limits research on its early bolting occurred mechanism. The use of high-throughput RNA-seq technology has recently been employed to generate large amounts of omics data of medicinal plants, such as *Angelica sinensis*^36^, *Panax ginseng*^37^, *Dictamnus dasycarpus*^38^ and so on. The research encompasses a range of areas including functional gene mining^12–14^, developmental mechanism research^16,17^, molecular marker development^18^, gene regulatory network construction and so on^20,21^. To address a gap in knowledge, we conducted a transcriptome sequencing of the roots of early bolting and non-bolting plants of *A. dahurica*. The RNA-seq analysis yielded 53,304 unigenes after de novo assembly, with an average length of 977 bp and an N50 length of 1,532 bp. These results demonstrate that the data obtained from *A. dahurica* has higher integrity and credibility.

Many studies have revealed that endogenous hormones directly regulate bolting and flowering, while cascading signals may also induce bolting and flowering^39^. Among the DEGs in the plant hormone signaling pathways(Supplementary Table S3), genes related to auxin-induced, such as *AUX6B*, and the auxin-responsive, *IAA26*, *SAUR32*, *SAUR36*, *SAUR61*, *SAUR67*, *SAUR72*, Gretchen Hagen 3, *GH3.1* were up-regulated in EB^23–25^. Kumar et al. have revealed that auxin and its corresponding receptors are necessary for the initiation of fowering and foral organ identity^26,27^. In our study, we observed a down-regulation of ethylene response transcription factors (ERFs), namely *RAV1*, *ERF13*, and *RAP2-7* in EB. It was earlier reported that ERFs are involved in the regulation of Arabidopsis bolting^28^. Down-regulation of *RAV1* in Arabidopsis leads to an early fowering phenotype^29^. *RAP2-7* negatively regulates the transition from vegetative to reproductive growth, results in a delay in fowering time^30^. Accordingly, the down-regulated expression of *RAV1* and *RAP2-7* in EB is consistent with its early bolting phenotype, as demonstrated by previous studies^29,30^. In the JA signaling pathway, the transcription factors *MYC2* and *MYC3* were found to be down-regulated in EB. These factors belong to the basic helix-loop-helix transcription factor family and act as high-level transcriptional regulators in the JA signaling pathway^31,32^. The studies of Wang et al. have shown that *MYC2* and *MYC3* are involved in JA-mediated fowering inhibition in Arabidopsis^33^. In conclusion, the study found that genes involved in multiple hormone signaling or metabolism pathways were differentially expressed between the two phenotypes, indicating that early bolting in *A. dahurica* is controlled by multiple hormones simultaneously.

The regulation of plant flowering is a complex process that involves the interaction of both internal and external factors. In recent years, significant progress has been made in understanding the control of flowering in different plants. The current understanding of the mechanism of flowering control involves six major pathways, namely photoperiod, vernalization, autonomous, temperature, gibberellin, and age pathways. These pathways form a complex network of genetic control channels that are both independent and interrelated^40^. The key genes of plant flowering regulation, such as *LFY*, *AP1*, *FLC*, *FT*, *SOC1*, etc., all play their roles through these pathways^41–43^. This study found that genes related to these pathways, namely *SOC1*, *FT*, *COL7*, and *VRN1*, were up-regulated in EB, while *FTIP1*, *GAIP*, and *CDF2* were down-regulated. Previous research by Yang et al. has demonstrated that the expression of *FT* and *SOC1* can initiate flowering by integrating signals from various pathways^44^. The *COL7* gene plays a crucial role in promoting flowering in onion by regulating the expression of two other genes, CONSTANS (*CO*) and FLOWERING LOCUS T (*FT*)^45,46^. Additionally, CDFs, a type of transcriptional regulator, act as repressors of *CO* transcription by binding to its regulatory regions^47^. This leads to down-regulated FT transcription and delayed flowering^48^. *VRN1* is a negative regulator of FLC, which can down-regulate the expression of FLC and promote the expression of downstream flowering integration genes *FT* and *SOC1*, thereby promoting bolting flowering^49^. Our results are consistent with those of previous studies, which suggest that we can use transcriptome sequencing technology to mine available differentially expressed genes, and lay a foundation for further understanding the molecular mechanisms of early bolting and flowering in *A. dahurica*.

The early bolting of *A. dahurica* had a significant impact on the root tissue, resulting in rapid lignification and hardening of the roots, and a decrease in effective components^4^. The study found significant differences in *4CL*, *COMT*, *HCT*, and *CSE* between early-bolting and non-bolting plants, with all four down-regulated in early-bolting plants. The synthesis of coumarins involves several key enzymes, including *4CL*, *COMT*, and *HCT*. When the expression of these enzymes is down-regulated, it can lead to a decrease in coumarin content^5,35^. In early bolting plants, researchers found that *ABCG22*, *ABCG29*, and *ABCG36* were up-regulated in the roots, while *MYB1* and *MYB63* were down-regulated. A study by Yao et al. demonstrated that *ABCG22*, *ABCG29*, and *ABCG36* can co-express with *MYB58* to jointly regulate the expression of genes involved in the synthesis of lignin monomers^50^. *MYB1* is known to negatively regulate the lignin branching pathway by down-regulating the expression of *EgCCR* and *EgCAD*, ultimately reducing lignification^51^. Interestingly, overexpression of *MYB63* in Arabidopsis leads to abnormal lignification of epidermis and mesophyll cells^52^, which contradicts the results obtained from down-regulating *MYB63* expression in this study. Further investigation is required to understand this inconsistency.

## Materials and methods

### Plant material

*A. dahurica* early-bolting and non-bolting plants were used for experimental purposes. These materials are allowed to be collected locally. They were collected on July 14 in the year 2021 at the study site in Suining City, Sichuan province, P. R. China (N 30°37 2032′, E 105°31′, elevation 286 m), and identified by Prof. Qingmao Fang. The voucher specimens have been preserved in the herbarium of the Sichuan Academy of Traditional Chinese Medicine Sciences under the identification number 510921210717001LY. There were three biological repetitions with 5 plants in each repetition. The roots of *A. dahurica* were sampled, rinsed with cold distilled water, and then immediately frozen in liquid nitrogen and stored at -80℃ in an ultra-low temperature freezer (Thermo, USA) until use. Concurrently, fresh materials (10 plants × three repetitions ) were collected for morphological investigation.

### Morphological analysis of *A. dahurica* root

The roots were initially measured for length(cm), diamete(mm), and fresh weigh(g). They were then dried at 60ºC until the weight no longer decreased, and the dry weight was measured. The root drying rate was calculated. The formula is as follows:

1 $$ {\text{Root}}\;{\text{ drying }}\;{\text{rate}}\left( \% \right) = \left( {{\text{Root}}\;{\text{ fresh }}\;{\text{weight}}/{\text{Root}}\;{\text{ dry }}\;{\text{weight}}} \right) \times {1}00 $$

### Determination of coumarins content

Coumarins content in dried *A. dahurica* roots was determined using the Agilent 1200 High Performance Liquid Chromatography (HPLC)^53^. Dried powder (0.5 g) was placed into aconical flask and mixed with 25 mL of 50% ethanol by ultrasonication (200 W, 50 HZ) for 1 h. After taking out to cool, add 50% ethanol to make up the loss of quality. The solution was shaken and centrifuged at 1500 r**·**min^22121^ for 5 min. The supernatant solution was thoroughly filtered through a 0.22 μm microporous membrane to obtain the test sample. All reference sub-stances (xanthotoxin, MUST-21012305; bergapten, MUST-20111610; imperatorin, MUST- 21,030,804; phellopterin, MUST-21060301; isoimperatorin, MUST-21090910; each 0.4 mg, HPLC > 98%) were mixed thoroughly with 10 mL of ethanol. Coumarins were detected by HPLC under these conditions: C18 chromatographic column (Agilent ZORBAX Eclipse Plius-C18, 250 mm × 4.6 mm, 5 μm); mobile phase of 0.1% methanol(A)-acetonitrile (B); an elution program consisting of 0–10 min, 10–25% B, 10–30 min, 25–45% B, 30–45 min 45–65% B, 45–471 min, 65–10%B, 47–50 min, 10% B; flow rate set to 1.5 mL/min; column temperature of 30 ℃; wavelength of 254 nm. All determinations were performed in triplicate for each sample.

### RNA extraction, cDNA library preparation and illumina sequencing

Total RNA was isolated from *A. dahurica* roots using the Plant RNA Kit (Aidlab Biotech, China) with three biological replicates of each sample. An RNA pool for each sample was prepared by combining equal amounts of RNA from the three replicates. Total RNA was analyzed by agarose gel electrophoresis for size and integrity. The quantification of total RNA was done with a Nanodrop 2000 (Thermo, USA). The sample for RNA sequencing was derived from the pooling of the RNA samples in two groups i.e. replicates isolated from the *A. dahurica* roots of early-bolting and non-bolting.

High-quality RNA libraries were sequenced on Illumina NovaSeq 6000 platform at Beijing Biomarker Technologies Co. Ltd. (Beijing, China).

### Transcriptome assembly and gene functional annotation

Raw reads obtained from sequencing were processed to obtain high-quality reads. Moreover, all reads were trimmed by using the Trimmomatic tool to remove low-quality reads and any adapter sequences if present^54^. The resultant high-quality reads of each sample underwent transcriptome assembly using Trinity 2.4.0 software with by using its default parameters^55^. Each unigene was functionally annotated using seven public databases: KEGG (http://www.genome.jp/kegg; Kyoto Encyclopedia of Genes and Genome), GO (http://geneontology.org; Gene Ontology), NT (ftp://ftp.ncbi.nlm.nih.gov/blast/db; NCBI nucleotide), NR (ftp://ftp.ncbi.nlm. nih.gov/blast/db; NCBI non-redundant protein sequence), KOG (http://www.ncbi.nlm.nih.gov/KOG; clusters of euKaryotic Orthologous Groups), Pfam (http://pfam.xfam.org; protein families), and SwissProt (http://ftp.ebi.ac.uk/pub/databases/swissprot; a manually annotated and reviewed protein sequence database). According to the NR annotation, the Blast2GO (https://www.blast2go.com) search was employed to derive the GO annotations of unigenes^56^.

### Differentially expressed genes (DEGs) analysis

The DEGs were screened by the Poisson distribution method^57^. The DEGs were selected by correcting the *P*-value of the difference test by multiple hypothesis testing. More specifically, the differential expression multiple of the gene among different samples was calculated according to the FPKM (Fragments Per Kilobase per Million) method. In general, DEGs are defined by default as those genes with an FDR (false discovery rate) < 0.01 and a FC (fold change) ≥ 2^58,59^.

### Validation of DEGs using qRT-PCR

Quantitative RT-PCR (qRT-PCR) was used to validate 8 candidate DEGs associated with early bolting and coumarin biosynthesis. Each qRT-PCR was implemented using the Power SYBR® Green PCR Master Mix (Roche) on a Quant-studioTM RealTime Detection System (Life Technologies, USA). The primers sequences, which were from other published articles^10,36,60^, can be found in Supplementary Table S4. The total volume of the reaction system was 20 μL, composed of 8 μL of sterile distilled deionized water, 10 uL of Power SYBR® Green Master Mix, 0.5 μL of the forward primer (10 μM), 0.5 μL of the reverse primer (10 μM), and 1 μL of cDNA. qRT-PCR was performed with the follow thermocycling parameters: 95 °C for 10 s, followed by 45 cycles of 95 °C for 10 s and 60 °C for 30 s. The 2^−ΔΔCt^ comparative threshold cycle (Ct) method was used to evaluate the relative expression levels of target genes^61^. The values reported represent the average of 3 biological replicates.

### Statistical analysis

Data were analyzed and plotted in WPS Office 11.1.0 (Kingsoft Corp., Beijing, China) and SPSS 20.0 (IBM Corp., Armonk, NY, USA) using analysis of variance (ANOVA) followed by Duncan’s significant difference test at *p* < 0.05 and *p* < 0.01. Experiments were performed with three repetitions.

### Ethical approval and consent to participate

The seeds were kindly provided by Suining Tiandiwang Chuanbaizhi Industry Co., Ltd., Suining, China. In this study, the experimental research and feld studies on plants, including collection of plant material, complied with relevant institutional, national, and international guidelines and legislation.

## Supplementary Information

Supplementary Information.

## Data Availability

Transcriptome datasets supporting the conclusions of this article are available in the NCBI SRA repository under the accession number SRR22096280. Reviewer link: https://www.ncbi.nlm.nih.gov/sra/?term=SRR22096280.
