# Supplementary material for: Analysis of the difference between early-bolting and non-bolting roots of Angelica dahurica based on transcriptome sequencing
Source: Sci Rep. 2023 May 15;13:7847. doi: 10.1038/s41598-023-34554-5 (PMC10185517; doi:10.1038/s41598-023-34554-5)

**Analysis of the difference between early-bolting and non-bolting roots of *Angelica dahurica* based on transcriptome sequencing**

Ping Wu, Xiaoyu Wang, Junxia Guo, Songli Zhang, Qingmiao Li*, Mei Zhang, Qingmao Fang, Bin Luo, Hongsu Wang & Weijin He

**SUPPLEMENTAL MATERIAL**

**Table S1：**Sutatistics of the annotations for *A. dahurica* unigenes from nine public databases.

| **Annotatio database** | **Annotated number** | **300<=length<1000** | **length>=1000** |
| --- | --- | --- | --- |
| COG annotation | 6,259 | 2,000 | 4,259 |
| GO annotation | 22,530 | 10,993 | 11,535 |
| KEGG annotation | 17,895 | 8,036 | 9,859 |
| KOG annotation | 14,693 | 6,646 | 8,047 |
| Pfam annotation | 18,786 | 7,591 | 11,195 |
| Swissprot annotation | 16,996 | 7,161 | 9,835 |
| TrEMBL annotation | 28,022 | 14,205 | 13,817 |
| eggNOG annotation | 22,697 | 10,783 | 11,914 |
| Nr annotation | 28,803 | 14,929 | 13,874 |
| All annotated | 29,401 | 15,466 | 13,933 |

**Table S2**:KEGG pathway analysis of *A. dahurica* transcriptome Unigenes

| pathway | pathway_id | Gene_number |
| --- | --- | --- |
| Glycolysis / Gluconeogenesis | ko00010 | 239 |
| Citrate cycle (TCA cycle) | ko00020 | 104 |
| Pentose phosphate pathway | ko00030 | 102 |
| Pentose and glucuronate interconversions | ko00040 | 353 |
| Fructose and mannose metabolism | ko00051 | 111 |
| Galactose metabolism | ko00052 | 290 |
| Ascorbate and aldarate metabolism | ko00053 | 254 |
| Fatty acid biosynthesis | ko00061 | 72 |
| Fatty acid elongation | ko00062 | 38 |
| Fatty acid degradation | ko00071 | 97 |
| Synthesis and degradation of ketone bodies | ko00072 | 8 |
| Cutin, suberine and wax biosynthesis | ko00073 | 56 |
| Steroid biosynthesis | ko00100 | 40 |
| Ubiquinone and other terpenoid-quinone biosynthesis | ko00130 | 63 |
| Oxidative phosphorylation | ko00190 | 264 |
| Photosynthesis | ko00195 | 125 |
| Photosynthesis - antenna proteins | ko00196 | 36 |
| Arginine biosynthesis | ko00220 | 54 |
| Purine metabolism | ko00230 | 161 |
| Caffeine metabolism | ko00232 | 5 |
| Pyrimidine metabolism | ko00240 | 91 |
| Alanine, aspartate and glutamate metabolism | ko00250 | 75 |
| Glycine, serine and threonine metabolism | ko00260 | 93 |
| Monobactam biosynthesis | ko00261 | 15 |
| Cysteine and methionine metabolism | ko00270 | 175 |
| Valine, leucine and isoleucine degradation | ko00280 | 84 |
| Valine, leucine and isoleucine biosynthesis | ko00290 | 18 |
| Lysine biosynthesis | ko00300 | 14 |
| Lysine degradation | ko00310 | 68 |
| Arginine and proline metabolism | ko00330 | 89 |
| Histidine metabolism | ko00340 | 39 |
| Tyrosine metabolism | ko00350 | 86 |
| Phenylalanine metabolism | ko00360 | 78 |
| Tryptophan metabolism | ko00380 | 83 |
| Phenylalanine, tyrosine and tryptophan biosynthesis | ko00400 | 69 |
| Benzoxazinoid biosynthesis | ko00402 | 14 |
| beta-Alanine metabolism | ko00410 | 82 |
| Taurine and hypotaurine metabolism | ko00430 | 10 |
| Phosphonate and phosphinate metabolism | ko00440 | 11 |
| Selenocompound metabolism | ko00450 | 30 |
| Cyanoamino acid metabolism | ko00460 | 57 |
| Glutathione metabolism | ko00480 | 107 |
| Starch and sucrose metabolism | ko00500 | 317 |
| N-Glycan biosynthesis | ko00510 | 76 |
| Other glycan degradation | ko00511 | 78 |
| Various types of N-glycan biosynthesis | ko00513 | 58 |
| Other types of O-glycan biosynthesis | ko00514 | 22 |
| Mannose type O-glycan biosynthesis | ko00515 | 4 |
| Amino sugar and nucleotide sugar metabolism | ko00520 | 378 |
| Polyketide sugar unit biosynthesis | ko00523 | 1 |
| Glycosaminoglycan degradation | ko00531 | 51 |
| Glycerolipid metabolism | ko00561 | 149 |
| Inositol phosphate metabolism | ko00562 | 135 |
| Glycosylphosphatidylinositol (GPI)-anchor biosynthesis | ko00563 | 30 |
| Glycerophospholipid metabolism | ko00564 | 152 |
| Ether lipid metabolism | ko00565 | 50 |
| Arachidonic acid metabolism | ko00590 | 41 |
| Linoleic acid metabolism | ko00591 | 36 |
| alpha-Linolenic acid metabolism | ko00592 | 78 |
| Sphingolipid metabolism | ko00600 | 132 |
| Glycosphingolipid biosynthesis - lacto and neolacto series | ko00601 | 3 |
| Glycosphingolipid biosynthesis - globo and isoglobo series | ko00603 | 14 |
| Glycosphingolipid biosynthesis - ganglio series | ko00604 | 38 |
| Pyruvate metabolism | ko00620 | 153 |
| Glyoxylate and dicarboxylate metabolism | ko00630 | 112 |
| Propanoate metabolism | ko00640 | 78 |
| Butanoate metabolism | ko00650 | 31 |
| C5-Branched dibasic acid metabolism | ko00660 | 5 |
| One carbon pool by folate | ko00670 | 23 |
| Carbon fixation in photosynthetic organisms | ko00710 | 120 |
| Thiamine metabolism | ko00730 | 47 |
| Riboflavin metabolism | ko00740 | 31 |
| Vitamin B6 metabolism | ko00750 | 16 |
| Nicotinate and nicotinamide metabolism | ko00760 | 37 |
| Pantothenate and CoA biosynthesis | ko00770 | 58 |
| Biotin metabolism | ko00780 | 18 |
| Lipoic acid metabolism | ko00785 | 5 |
| Folate biosynthesis | ko00790 | 32 |
| Porphyrin and chlorophyll metabolism | ko00860 | 67 |
| Terpenoid backbone biosynthesis | ko00900 | 63 |
| Indole alkaloid biosynthesis | ko00901 | 5 |
| Monoterpenoid biosynthesis | ko00902 | 20 |
| Limonene and pinene degradation | ko00903 | 18 |
| Diterpenoid biosynthesis | ko00904 | 101 |
| Brassinosteroid biosynthesis | ko00905 | 35 |
| Carotenoid biosynthesis | ko00906 | 53 |
| Zeatin biosynthesis | ko00908 | 55 |
| Sesquiterpenoid and triterpenoid biosynthesis | ko00909 | 90 |
| Nitrogen metabolism | ko00910 | 49 |
| Sulfur metabolism | ko00920 | 49 |
| Phenylpropanoid biosynthesis | ko00940 | 202 |
| Flavonoid biosynthesis | ko00941 | 89 |
| Anthocyanin biosynthesis | ko00942 | 13 |
| Isoflavonoid biosynthesis | ko00943 | 18 |
| Flavone and flavonol biosynthesis | ko00944 | 13 |
| Stilbenoid, diarylheptanoid and gingerol biosynthesis | ko00945 | 44 |
| Isoquinoline alkaloid biosynthesis | ko00950 | 59 |
| Tropane, piperidine and pyridine alkaloid biosynthesis | ko00960 | 48 |
| Betalain biosynthesis | ko00965 | 19 |
| Glucosinolate biosynthesis | ko00966 | 8 |
| Aminoacyl-tRNA biosynthesis | ko00970 | 98 |
| Biosynthesis of various secondary metabolites - part 2 | ko00998 | 4 |
| Biosynthesis of unsaturated fatty acids | ko01040 | 52 |
| Carbon metabolism | ko01200 | 437 |
| 2-Oxocarboxylic acid metabolism | ko01210 | 77 |
| Fatty acid metabolism | ko01212 | 129 |
| Biosynthesis of amino acids | ko01230 | 346 |
| ABC transporters | ko02010 | 128 |
| Ribosome biogenesis in eukaryotes | ko03008 | 129 |
| Ribosome | ko03010 | 418 |
| RNA transport | ko03013 | 324 |
| mRNA surveillance pathway | ko03015 | 209 |
| RNA degradation | ko03018 | 202 |
| RNA polymerase | ko03020 | 144 |
| Basal transcription factors | ko03022 | 56 |
| DNA replication | ko03030 | 88 |
| Spliceosome | ko03040 | 374 |
| Proteasome | ko03050 | 59 |
| Protein export | ko03060 | 82 |
| Base excision repair | ko03410 | 56 |
| Nucleotide excision repair | ko03420 | 99 |
| Mismatch repair | ko03430 | 79 |
| Homologous recombination | ko03440 | 141 |
| Non-homologous end-joining | ko03450 | 8 |
| MAPK signaling pathway - plant | ko04016 | 401 |
| Phosphatidylinositol signaling system | ko04070 | 125 |
| Plant hormone signal transduction | ko04075 | 496 |
| Ubiquitin mediated proteolysis | ko04120 | 206 |
| Sulfur relay system | ko04122 | 13 |
| SNARE interactions in vesicular transport | ko04130 | 41 |
| Autophagy - other | ko04136 | 48 |
| Protein processing in endoplasmic reticulum | ko04141 | 460 |
| Endocytosis | ko04144 | 311 |
| Phagosome | ko04145 | 114 |
| Peroxisome | ko04146 | 141 |
| Plant-pathogen interaction | ko04626 | 689 |
| Circadian rhythm - plant | ko04712 | 93 |

**Table S3**:DEGs associated with early bolting and biosynthesis of coumarin and lignin metabolism.

| **Number** | **type** | **Gene name** | **Protein name** | **NB-**  **FPKM** | **EB-**  **FPKM** | **Regulation** |
| --- | --- | --- | --- | --- | --- | --- |
| 1 | floral organ development | *AGL62* | Agamous-like MADS-box protein AGL62 | 0.13 | 29.65 | up |
| 2 |  | *AGL8* | Agamous-like MADS-box protein AGL8 | 13.33 | 37.43 | up |
| 3 |  | *AP1* | Agamous-like MADS-box protein AP1 | 0.98 | 215.62 | up |
| 4 | Genes involved in plant hormone metabolism pathways | *ABAH2* | Abscisic acid 8'-hydroxylase | 0 | 2.72 | up |
| 5 |  | *CKX1* | Cytokinin dehydrogenase 1 | 2.11 | 8.84 | up |
| 6 |  | *CKX6* | Cytokinin dehydrogenase 6 | 1.26 | 8.04 | up |
| 7 |  | *CKX7* | Cytokinin dehydrogenase 7 | 0.35 | 3.5 | up |
| 8 |  | *NCED2* | 9-cis-epoxycarotenoid dioxygenase NCED2, chloroplastic OS=Solanum lycopersicum OX=4081 GN=NCED2 PE=2 SV=1 | 0.31 | 3.9 | up |
| 9 |  | *AUX6B* | Auxin-induced protein AUX | 0 | 4.13 | up |
| 10 |  | *ERF13* | Ethylene-responsive transcription factor 13 | 18.335 | 2.17 | down |
| 11 |  | *RAP2-7* | Ethylene-responsive transcription factor RAP2-7 | 18.63 | 2.61 | down |
| 12 |  | *RAV1* | AP2/ERF and B3 domain-containing transcription factor RAV1 | 18.73 | 0.24 | down |
| 13 |  | *GASA1* | Gibberellin-regulated protein 1 | 496.3 | 5.24 | down |
| 14 |  | *GASA11* | Gibberellin-regulated protein 11 | 361 | 68.5 | down |
| 15 |  | *GASA14* | Gibberellin-regulated protein 14 | 12.8 | 0.31 | down |
| 16 |  | *GH3.1* | Probable indole-3-acetic acid-amido synthetase GH3.1 | 3.49 | 40.05 | up |
| 17 |  | *IAA14* | Auxin-responsive protein IAA14 | 20.16 | 2.41 | down |
| 18 |  | *IAA26* | Auxin-responsive protein IAA26 | 4.35 | 29.93 | up |
| 19 |  | *IAA27* | Auxin-responsive protein IAA27 | 26.66 | 4.21 | down |
| 20 |  | *SAU32* | Auxin-responsive protein SAUR32 | 27.5 | 201.6 | up |
| 21 |  | *SAU36* | Auxin-responsive protein SAUR36 | 6.6 | 107.38 | up |
| 22 |  | *SAU40* | Auxin-responsive protein SAUR40 | 5.66 | 0.19 | down |
| 23 |  | *SAU61* | Auxin-responsive protein SAUR61 | 1.27 | 5.77 | up |
| 24 |  | *SAU67* | Auxin-responsive protein SAUR67 | 0 | 5.24 | up |
| 25 |  | *SAU72* | Auxin-responsive protein SAUR72 | 2.4 | 17.76 | up |
| 26 |  | *SAU76* | Auxin-responsive protein SAUR76 | 18.7 | 2.08 | down |
| 27 |  | *MYC2* | transcription factor MYC2 | 21.75 | 3.38 | down |
| 28 |  | *MYC3* | Transcription factor MYC3 | 12.33 | 1.45 | down |
| 29 | photoperiodic pathway | *CDF2* | Cyclic dof factor 2 | 1.05 | 0.04 | down |
| 30 |  | *COL13* | Zinc finger protein CONSTANS-LIKE 13 | 41.7 | 12.98 | down |
| 31 |  | *COL5* | Zinc finger protein CONSTANS-LIKE 5 | 1.28 | 0 | down |
| 32 |  | *COL7* | Zinc finger protein CONSTANS-LIKE 7 | 13.27 | 75.45 | up |
| 33 |  | *EFM* | Myb family transcription factor EFM | 9.37 | 0.91 | down |
| 34 |  | *FTIP1* | FT-interacting protein 1 | 6.53 | 1.15 | down |
| 35 |  | *HD3A* | Flowering locus T | 0 | 6.41 | up |
| 36 |  | *SOC1* | MADS-box protein SOC1 | 0.59 | 29.85 | up |
| 37 |  | *MIP1A* | B-box domain protein 30 | 35.2 | 3.08 | down |
| 38 |  | *MIP1B* | B-box domain protein 31 | 21.74 | 1.2 | down |
| 39 | vernalization pathway | *VRN1* | B3 domain-containing transcription factor VRN1 | 0 | 4.73 | up |
| 40 | gibberellin pathway | *GA20OX1* | Gibberellin 20 oxidase 1 | 0.12 | 19.37 | up |
| 41 |  | *GA20OX2* | Gibberellin 20 oxidase 2 | 0 | 4.09 | up |
| 42 |  | *GA2OX1* | Gibberellin 2-beta-dioxygenase 1 | 86.95 | 1.27 | down |
| 43 |  | *GA2OX5* | Gibberellin 2-beta-dioxygenase 5 | 0 | 6.42 | up |
| 44 |  | *GA2OX6* | Gibberellin 2-beta-dioxygenase 6 | 4.61 | 1 | down |
| 45 |  | *GAIP* | DELLA protein GAIP | 3.85 | 0.47 | down |
| 46 | biosynthesis of coumarin metabolism | *4CL* | 4-coumarate--CoA ligase | 8.08 | 0.23 | down |
| 47 |  | *COMT* | Caffeic acid 3-O-methyltransferase | 40.21 | 4.023 | down |
| 48 |  | *CSE* | Caffeoylshikimate esterase | 7.17 | 3.49 | down |
| 49 |  | *HCT* | Shikimate O-hydroxycinnamoyltransferase | 13.38 | 1.54 | down |
| 50 | biosynthesis of lignin metabolism | *ABCG22* | ABC transporter G family member 22 | 0.18 | 12.61 | up |
| 51 |  | *ABCG36* | ABC transporter G family member 36 | 11.94 | 58.82 | up |
| 52 |  | *MYB1* | Transcription factor MYB1 | 57.25 | 2.96 | down |
| 53 |  | *MYB63* | Transcription factor MYB63 | 3.17 | 0.26 | down |

**Table S4：**Gene and primers used for the qRT-PCR.

| **Number** | **Genes** | **Primer pairs** |
| --- | --- | --- |
| 1 | *actin* | F:5'-GATTCCGTTGCCCTGAGGTTCTG -3' |
|  |  | R: 5'-ACCACCACTGAGCACTATGTTTCC-3' |
| 2 | *4CL* | F:5'-CAACTCATGGAAGCTGACGAAT-3' |
|  |  | R: 5'- ATTCTCGCCATCAACTTGTTGT-3' |
| 3 | *HCT* | F:5'-CTTAGCTGGTCCCACATTCTTG-3' |
|  |  | R: 5'-AAGAAGGGAGTTTGCAGGTTTC-3' |
| 4 | *CSE* | F:5'-CCTAAACCGAAAGCAGGAGAAG-3' |
|  |  | R: 5'-GAATCCCAAACCATTCCCTCAG-3' |
| 5 | *COMT* | F:5'-AGTTACATTGCCGACGAAGATG-3' |
|  |  | R: 5'-CCCTCAAGCAGTGCATCTTTTA-3' |
| 6 | *RAP2-7* | F: 5'-CGCACCACAATCTGGACCTTAGC-3' |
|  |  | R: 5'-GAGCAGGAGCAAACCTCTCAACC-3' |
| 7 | *RAV1* | F: 5'-TCGTGTCGGGTCGGGTTGTG-3' |
|  |  | R: 5'-ACCCCATCTCCCTCGTTCTCTTTC-3' |
| 8 | *MYC2* | F:5'-GCCTGCGAATGGGAGAGAAGAAC-3' |
|  |  | R: 5'-ATTAGGCACCACAACACGAAGAGC-3' |
| 9 | *COL5* | F: 5'-AGAGAACTGATGGAAATCGGCA-3' |
|  |  | R:5'- CGTTATGGCCAACACCAAAGAG-3' |

**Figure S1：** Length distribution of *A. dahurica* unigenes.


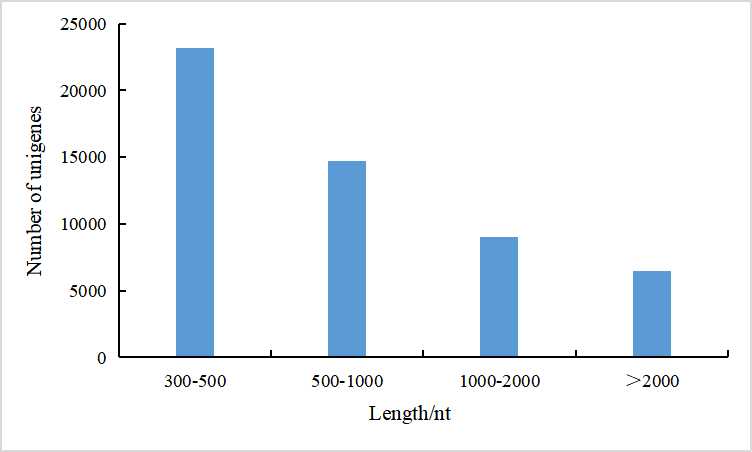


**Figure S2:**Homologous species distribution (determined using the NR database).


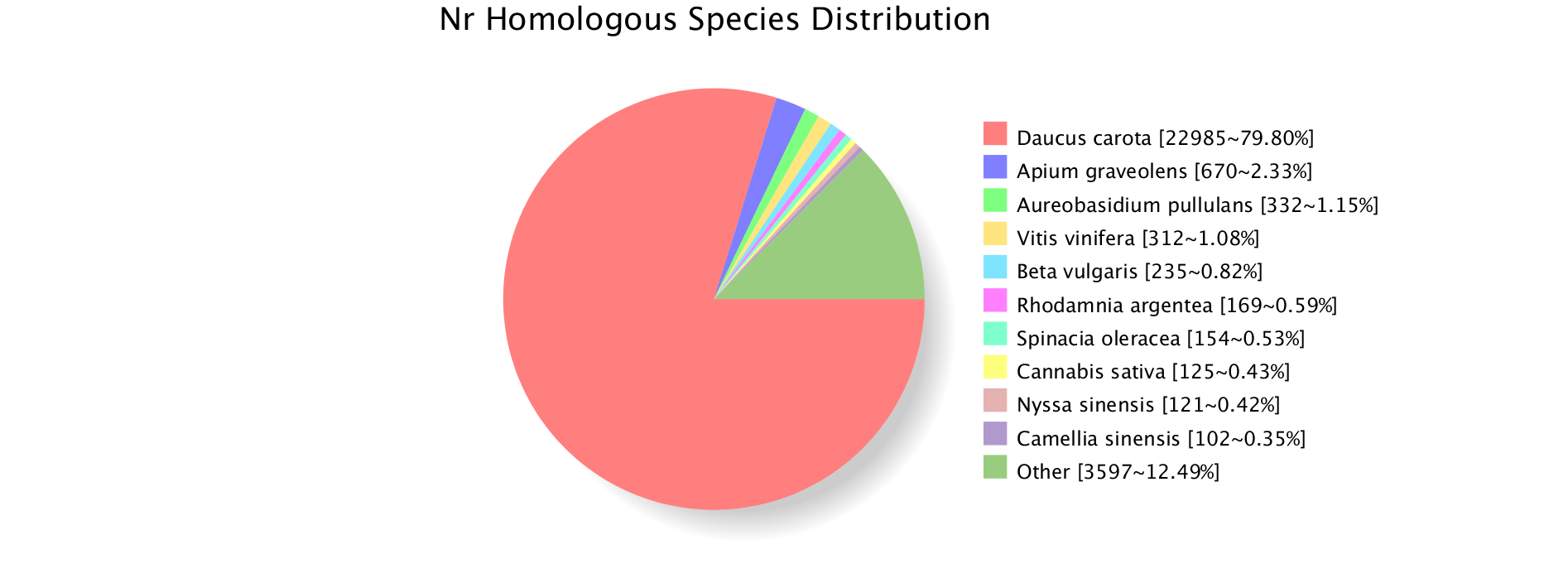
:

**Figure S3:**Functional classification in the COG pathways.


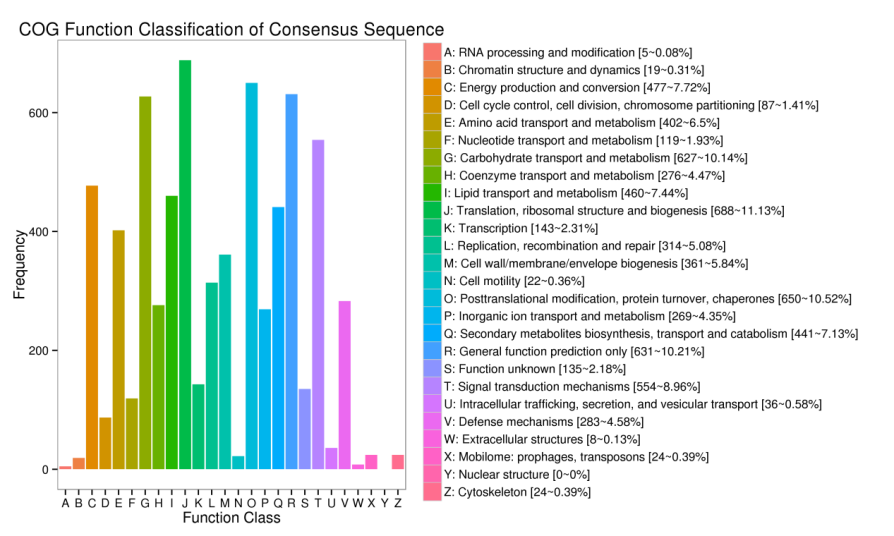


**Figure S4:** Functional classification in the GO pathways.


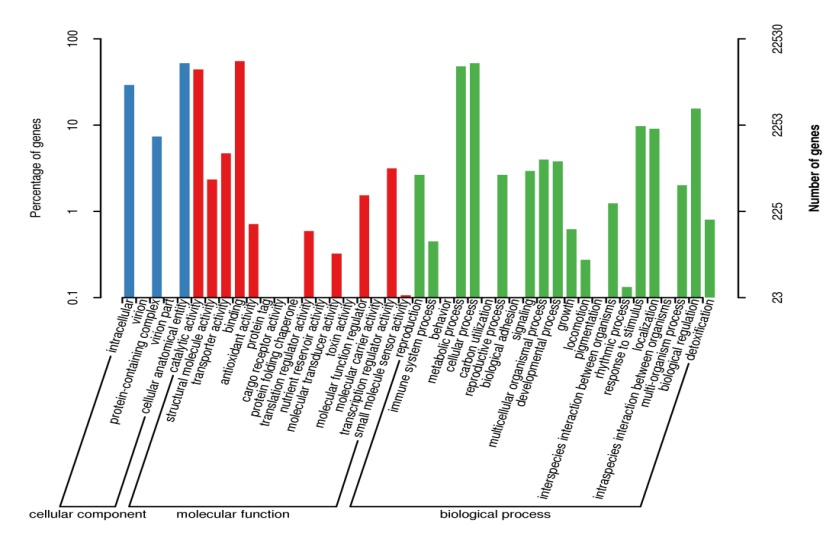


**Figure S5:** Enriched GO Terms of DEGs generated from the EB and NB group


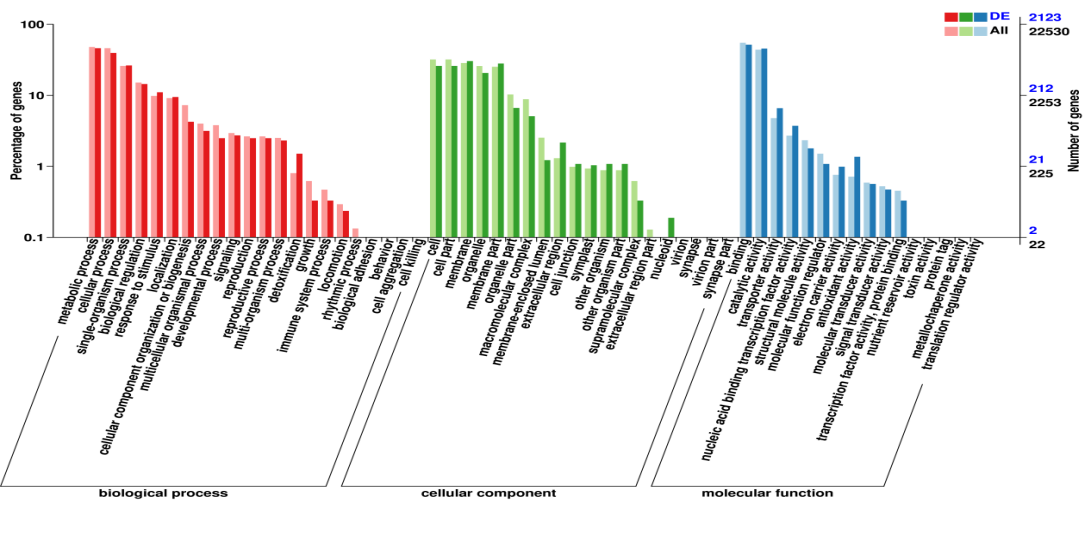


**Figure S6:**KEGG enrichment of DEGs


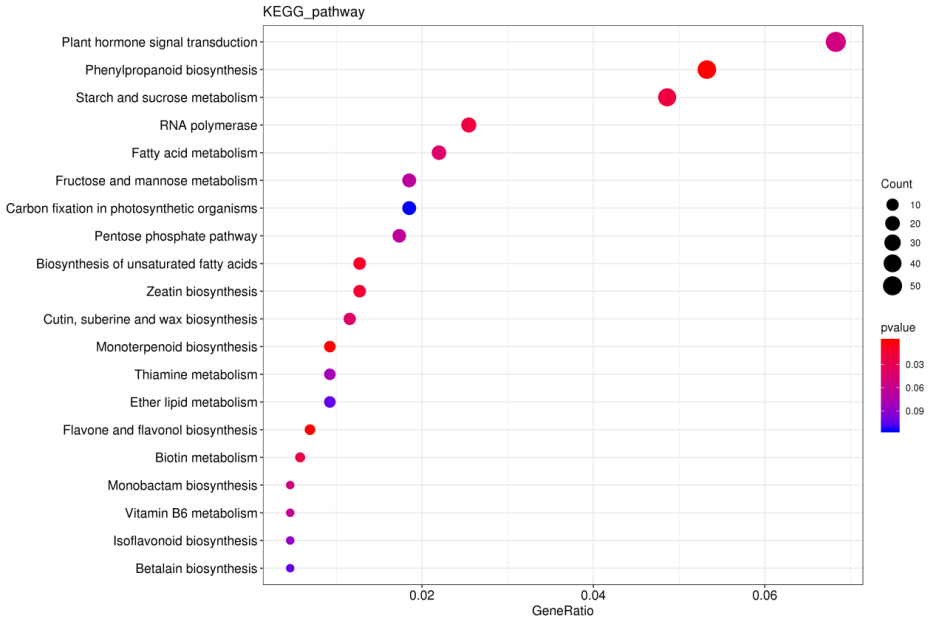

Supplement: Supplementary file 1 — Supplementary Information. [file 41598_2023_34554_MOESM1_ESM.docx]
